# Mechanistic Insight into Antimicrobial and Antioxidant Potential of *Jasminum* Species: A Herbal Approach for Disease Management

**DOI:** 10.3390/plants10061089

**Published:** 2021-05-28

**Authors:** Acharya Balkrishna, Akansha Rohela, Abhishek Kumar, Ashwani Kumar, Vedpriya Arya, Pallavi Thakur, Patrik Oleksak, Ondrej Krejcar, Rachna Verma, Dinesh Kumar, Kamil Kuca

**Affiliations:** 1 Patanjali Herbal Research Department, Patanjali Research Institute, Haridwar 249405, Uttarakhand, India; acharya.balkrishnapri@prft.in (A.B.); akansha.rohela@prft.co.in (A.R.); abhishekkumar@prft.co.in (A.K.); vedpriya.arya@prft.in (V.A.); pallavi.thakur@prft.co.in (P.T.); 2 Department of Chemistry, Faculty of Science, University of Hradec Kralove, 50003 Hradec Kralove, Czech Republic; patrik.oleksak@uhk.cz; 3 Center for Basic and Applied Science, Faculty of Informatics and Managemet, University of Hradec Kralove, 50003 Hradec Kralove, Czech Republic; ondrej.krejcar@uhk.cz; 4 School of Biological and Environmental Sciences, Shoolini University of Biotechnology and Management Sciences, Solan 173229, India; 5 School of Bioengineering and Food Technology, Shoolini University of Biotechnology and Management Sciences, Solan 173229, India; chatantadk@yahoo.com; 6 Biomedical Research Center, University Hospital in Hradec Kralove, Sokolska 581, 50005 Hradec Kralove, Czech Republic

**Keywords:** *Jasminum* species, antimicrobial, antioxidants, mechanistic insight, reactive oxygen species

## Abstract

Drug resistance among microbial pathogens and oxidative stress caused by reactive oxygen species are two of the most challenging global issues. Firstly, drug-resistant pathogens cause several fatalities every year. Secondly aging and a variety of diseases, such as cardiovascular disease and cancer, are associated with free radical generated oxidative stress. The treatments currently available are limited, ineffective, or less efficient, so there is an immediate need to tackle these issues by looking for new therapies to resolve resistance and neutralize the harmful effects of free radicals. In the 21st century, the best way to save humans from them could be by using plants as well as their bioactive constituents. In this specific context, *Jasminum* is a major plant genus that is used in the Ayurvedic system of medicine to treat a variety of ailments. The information in this review was gathered from a variety of sources, including books, websites, and databases such as Science Direct, PubMed, and Google Scholar. In this review, a total of 14 species of *Jasminum* have been found to be efficient and effective against a wide variety of microbial pathogens. In addition, 14 species were found to be active free radical scavengers. The review is also focused on the disorders related to oxidative stress, and it was concluded that *Jasminum grandiflorum* and *J. sambac* normalized various parameters that were elevated by free radical generation. Alkaloids, flavonoids (rutoside), terpenes, phenols, and iridoid glucosides are among the main phytoconstituents found in various *Jasminum* species. Furthermore, this review also provides insight into the mechanistic basis of drug resistance, the generation of free radicals, and the role of *Jasminum* plants in combating resistance and neutralizing free radicals.

## 1. Introduction

Emerging infections and the rise in antibiotic resistance among pathogens have been the major challenges that will endanger society’s health today. Worldwide, millions of deaths are attributed to microbial diseases. In 2013, a total of 9.2 million deaths due to microbial infections were reported [1,2]. The occurrence of drug resistance has resulted in a decline in the efficacy and ineffectiveness of existing antibiotics [3,4]. Recently, several studies have mainly focused primarily on seeking promising ways to solve these challenges, looking for new antimicrobials, as well as modification of existing ones. Interestingly, as the safest source to obtain various medicines, medicinal plants are preferred, as per the World Health Organization [5].

Plants and their bioactive compounds could be one of the most promising antimicrobial agents to fight resistance in the current scenario. They are pioneers in the discovery of new medicines, used directly or as a precursor for the synthesis of bioactive constituents. Phytochemicals have exerted antimicrobial action by different mechanisms against a wide variety of pathogens (susceptible and resistant). In plants, significant quantities of secondary metabolites are found, such as tannins, alkaloids, phenolics, and flavonoids [6,7]. A large number of medicinal plants have been shown to be effective in treating bacterial infections [8]. Researchers around the world have already investigated and continue to explore a variety of plants for their antimicrobial activity. For instance, *Osyris auriculata* and *Ficus auriculata* were found to be active towards *Escherichia coli* and *Salmonella typhimurium* [9].

On the other hand, under metabolic conditions, oxygen, an efficient molecule, can create free radicals; both are important and essential in the biochemical process and in redox reactions [10,11]. A number of oxygen free radicals are grouped under reactive oxygen species (ROS). However, ROS are highly unstable, attacking nucleic acids, lipids, and proteins, initiating a chain reaction, and playing a part in the etiology of major human diseases [12,13]. In addition, ROS generation is also associated with cancer, and cardiovascular disorders [14]. In regard to this, plant phytochemicals provide significant protection against free radicals as they neutralize their harmful effects. For instance, *Prunus domestica*, *Syzygium cumini*, *Rubus ellipticus*, and *Prunus armeniaca* fruits were found to be enriched with antioxidants [15]. Interestingly, medicinal plants could be seen as a precious gift from nature to humans [16].

*Jasminum* in the *Oleaceae* family are an important group of flowering plants, commonly cultivated for their aromatic flowers [17]. There are 197 taxonomically recognized species (spp.) of the genus *Jasminum* in the world [18]. *Jasminum* plants have been recommended against intestinal worms and venereal diseases [19,20]. Notably, in the aspect of medicine and pharmaceutical sectors, almost all parts of the plant are important [17]. Flowers are used for treating vesicles, ulcers, skin diseases, boils, and eye disorders, while leaves are used against breast tumors [20]. Traditionally, many members of the *Jasminum* genus have been used medicinally. For instance, *J. officinale* exhibits various therapeutic properties, viz., depurative, analgesic, diuretic, antiseptic, expectorant, anti-depressant, and sedative [21]. It is used by various tribals for treating gastrointestinal disorders, cough, pyrexia, eye inflammation, and also against irregular menstruation [22]. Likewise, *J. grandiflorum* is recommended against cough, hysteria, uterine ailments, and partum problems [23]. Subsequently, *J. sambac* is expectorant, analgesic, antiseptic, aphrodisiac, anti-depressant, and sedative [24]. Its flowers are taken orally by the Meitei community to treat piles [25]. Furthermore, a decoction of its roots (3 g) mixed with honey (l0 g) is taken twice daily in the treatment of amenorrhoea [26]. This review focuses on evaluating the antimicrobial and antioxidant ability of *Jasminum* spp., keeping in mind the emergence of antibiotic resistance and oxidative stress induced by ROS. The first half of the review sheds light on their antimicrobial potential and mechanistic viewpoint of drug resistance, whereas the second half demonstrates the antioxidant potential, the effectiveness of plants against disorders associated with oxidative stress, and the mechanistic basis for free radical neutralization by *Jasminum* spp.

## 2. Search Strategy

Different databases like Science Direct, PubMed, and Google Scholar have been explored in this review with various keywords such as *Jasminum* species, antimicrobial, antibacterial, antifungal, antioxidant activity, reactive oxygen species, oxidative stress, phytochemistry, bacterial and antifungal drug resistance. The compilation of literature was carried out between 1 December 2020 and 1 February 2021. In order to maintain quality, only full length, original, and English language papers from Web of Science and Scopus indexed peer-reviewed journals have been included in this study. Further, papers with more than 5 citations are also included in some cases. The current review was compiled based on 100 studies, 34 review papers, 4 website reports, and 5 books that were published between 1971 and 2021.

## 3. Distribution of Genus *Jasminum*

**Figure 1:**
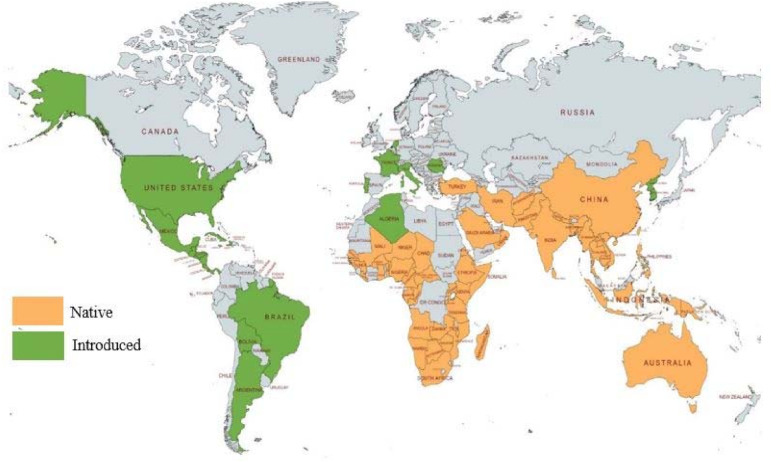
Global distribution of genus *Jasminum* (Created with mapchart.net).

The native range of the genus *Jasminum* covers tropical, subtropical Old World to Central China and the Pacific region (Figure 1). Further, they have been introduced into Europe, the Caribbean region, South and Central America, and the U.S.A. [18].

## 4. Antimicrobial Profile of *Jasminum* spp.

A total of 14 *Jasminum* spp. have been documented for their anti-microbial activity against Gram positive and negative bacterial strains, and fungal pathogens. In terms of efficacy against a wide variety of bacterial pathogens and minimal antifungal activity, all of the *Jasminum* plants are extremely encouraging.

A fraction of acetone extract from the leaves of *Jasminum azoricum* has shown anti-*Staphylococcus aureus* activity with the highest inhibition zone (30 mm at 30 mg/mL) among all the 14 species studied, whereas methanolic extract of *Jasminum syringifolium* leaves exhibited a 22.67-mm inhibition zone against *Shigella flexneri* (Table 1). Further, jatamansone extract from leaves of *Jasminum brevilobum* has shown the lowest minimum inhibitory concentration (MIC 0.05 μg/mL) against *Staphylococcus aureus* among all the studied species, whereas, it showed the highest MIC against *Escherichia coli* (MIC 0.07 μg/mL).

Compared to Gram negative ones, the impact is more obvious in the case of Gram positive pathogens. The difference in susceptibility between Gram negative positive strains is due to structural dissimilarities and composition of membranes [44,45]. Thus, from Table 1, it was concluded that, among other species, *Jasminum azoricum* and *Jasminum brevilobum* were found to be the most active species.

However, most of the plant species displayed no fungal activity except for a few species. Methanol extract of *Jasminum grandiflorum* subsp. *floribundum* has demonstrated anti-fungal activity against *Candida albicans* with a zone of inhibition of 22 mm. Meanwhile, essential oil from flowers extract of *Jasminum officinale* has shown anti-fungal activity against the *Trichosporon ovoides* with MIC 3.1 μg/mL.

## 5. Role of *Jasminum* Plants in Combating Resistance

In the 21st century, antibiotic resistance has become a serious public health concern. Bacterial and fungal strains constantly develop new ways through various unknown/undescribed mechanisms to adapt and withstand the biostatic or lethal effects of antibiotics [46,47]. Key factors leading to resistance include misuse and abuse of antimicrobials over decades, inadequate laboratory resources, and poor surveillance. In particular, their introduction to human as well as veterinary medicine contributed a lot in this regard [48,49,50,51].

### 5.1. Bacterial Antibiotic Resistance

**Figure 2:**
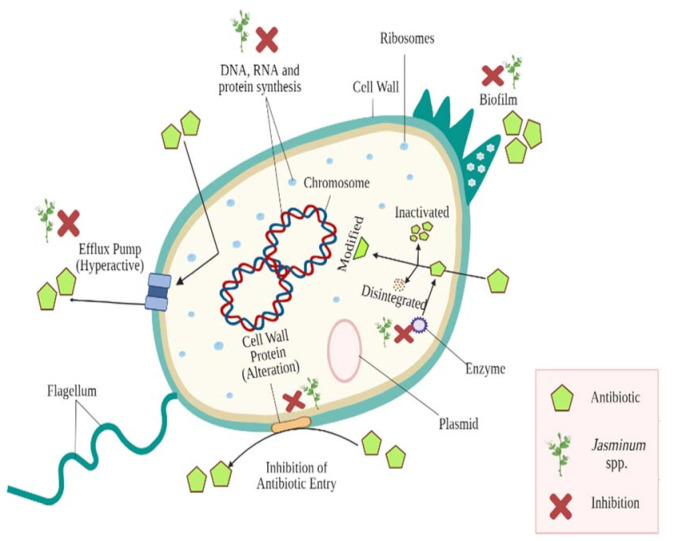
Major antibiotic resistance mechanisms evolved among bacterial strains and impact of *Jasminum* spp. against them (Created using Biorender.com).

The antibacterial drug’s mechanism usually involves degradation of the bacterial membrane, and an inhibitory effect on biosynthesis of the cell wall and synthesis of nucleic acid [52,53,54]. Bacterial strains have number of well-differentiated mechanisms by which they survive and develop antibiotic resistance [50,55,56]. The mechanistic basis of resistance includes numerous paths, such as molecular target alteration, efflux pumps’ overexpression, formation of biofilm, antibiotic degradation or modification, enzyme mediated destruction, and modification of bacterial target structures [49,50,57,58,59,60,61,62]. These mechanisms are shown in Figure 2. The mechanisms mentioned above assist bacteria to withstand pressure of antibiotic selection. Hydrolysis, functional group transfer, and structural modifications of antibiotics can be caused by a wide range of bacterial enzymes, thereby limiting their effectiveness. The standard process of making the β-lactam class of antibiotics ineffective is hydrolysis [63,64,65]. In bacterial strains, efflux pumps constitute the major resistance mechanism as their hyperactivity in resistant strains efflux antimicrobials outside the cell, reducing their concentration and thereby rendering them inefficient [59,66]. These mechanisms might be innately encoded within bacterial chromosome and through random mutations in chromosomal genes [56]. In addition, plasmids containing resistance genes can confer antimicrobial resistance [46].

### 5.2. Antibiotic Resistance in Fungi

Fungal infections seem to be a critical threat in clinical research over the past few decades, with immune-compromised individuals becoming readily susceptible. In particular, fungal infections are commonly linked to higher mortality [67]. Moreover, in healthy populations with an increased occurrence of fungal pathogens such as *Aspergillus fumigatus*, there are increasing indications of fungal infections, rendering fungi a potent threat*. Candida auris*, for example, made headlines as an emerging pandrug-resistant microorganism to effective antifungal drugs [67]. Four major groups, namely azoles, polyenes, echinocandins, and allylamines constitute currently available antifungal drugs. Ergosterol (polyenes), its biosynthetic route (allylamines and azoles), and β-glucan synthesis (echinocandins) are among the targets of many antifungal drugs; unfortunately, these protein molecules can trigger the emergence of resistance [68]. Polyenes and azoles hinder biosynthesis of ergosterol, a sterol found in fungal membranes. However, their use in humans is confined because of their toxicity that affects mammalian cholesterol which has structural similarities with ergosterol of fungal strains [68]. Considering that drug options are minimal, studies examining resistance mechanisms to current antifungals are valuable.

**Figure 3:**
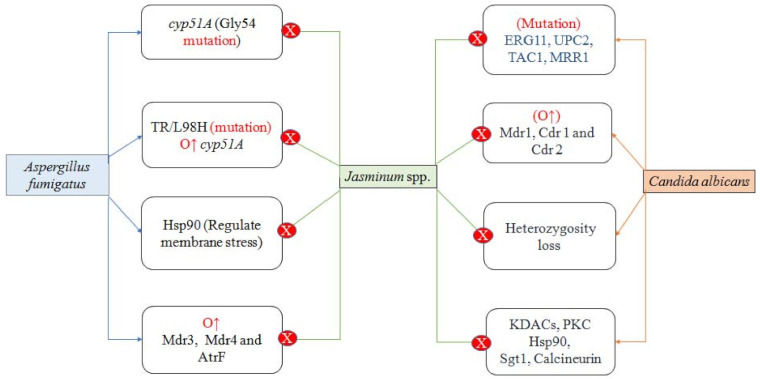
Markers of antifungal resistance in *Aspergillus fumigatus* and *Candida albicans* and protective role of *Jasminum* plants against the same. Overexpression (O↑); Lanosterol 14-α demethylase (ERG11); Transcription factor (UPC2, MRR1), Hsp90 (Heat shock protein-90), Cytochrome P450 14α-sterol demethylases (*Cyp51*); Multidrug resistant (MDR); Complementarity-determining regions (CDRs), Lysine deacetylases (*KDAC*); Protein kinase C (PKC), ATP Binding Cassette transporter gene (AtrF); Transcriptional activator of CDR genes (TAC1).

In this study, azoles resistance mechanism of *Candida albicans* and *Aspergillus fumigatus* have been reviewed (Figure 3). Azole resistance may develop through a variety of mechanisms, including drug target overexpression or modification, drug transporter upregulation, or cellular changes that minimise drug toxicity or allow tolerance to drug-induced stress [69,70].

The resistance mechanism of *Candida albicans* involves mutations in lanosterol 14-α demethylase (ERG11); transcription factor UPC2, TAC1 (transcriptional activator of CDR genes), and MRR1 (transcription factor); heterozygosity loss; and overexpression of Cdr1, Cdr2 (*Candida albicans* multidrug resistant protein), and Mdr1 (multidrug resistant protein 1). A number of stress-response pathway mediators (Hsp90 (Heat shock protein-90), Sgt1, calcineurin, KDACs (lysine deacetylases), PKC (protein kinase C) can also contribute to resistance. On the other hand, *Aspergillus fumigatus* resistance is related to mutation at Gly54 (position) in the cyp51A gene, while overexpression of cyp51A is due to TR/L98H mutations. Additionally, there is overexpression of ATP Binding Cassette (ABC) transporter gene (AtrF), Mdr3, and Mdr4. Similar to *Candida albicans,* Hsp90 is major contributing stress-response pathway mediator [70,71,72,73].

### 5.3. Protective Role of Jasminum Species

The activity of plants is related to their bioactive composition. Although the mechanisms of action of plant bioactive substances (PBS) are not clear, they are assumed to intervene with cell membrane organisation, leading to decreased membrane potential and lower levels of synthesis of ATP. The addition of PBS to the medium induces cellular membrane permeability, chelation of metal ions, and disruption of membrane-bound ATPase activity that alters the bacteria’s physiological state and leads to the death of the bacterial strain [74,75]. PBS is capable of acting on many bacterial resistance production targets. They play an important role as drug-inactivating enzyme inhibitors, as well as being involved in inhibition of efflux pump over-expression. In addition, they inhibit synthesis of protein and DNA and also exhibit anti-biofilm activity (Figure 2). Several other researchers have expressed similar views. [59,60].

There are reports that carvacrol, thymol, as well as eugenol and catechins are reported for ATP depletion through membrane structure degradation leading to discharge of cellular components [76,77,78]. In addition, tea tree oil, consisting of monoterpenes, terpenes, sesquiterpenes, 1,8-cineol, alpha-terpineol, and terpinen-4-ol, is capable of interfering with the permeability of the membrane, destroying the cell membrane and obstructing cell development, causing cell death in resistant microbes (*Staphylococcus aureus, Escherichia coli,* and *Candida albicans* [79]).

A brief overview of the different phytoconstituents of *Jasminum* spp. is compiled here. In continuation, root bark of *Jasminum abyssinicum* contains flavonoids, glycosides, saponins, secoiridoid glucosides, terpenoids, and triterpenes [80,81], while leaves of *Jasminum angustifolium* contain alkaloids, flavonoids, phenolics, saponins, sterols, tannins, and terpenoids [82]. Similarly, *Jasminum angustifolium* var. *sessiliflorum* contains alkaloids, glycosides, phenols, quinones (anthraquinones), saponins, steroids, terpenoids, and tannins [83,84]. Moreover, leaves as well as flowers of *Jasminum arborescens* contain alkaloids (quinines), flavonoids, phenols, saponins, and terpenoids [20].

*Jasminum auriculatum* contains flavonoids, phenolics, and terpenoids [85]. Its flowers contain alkaloids, essential oil, flavonoids, glycosides, phenolic acid (salicylic acid), sterols, and tannins [86]. Leaves of *Jasminum azoricum* contain alkaloids, coumarins, flavonoids (kaempferol, quercetin, rutoside), glycosides, iridoid glucosides (azoricin, sambacin), polyphenols, quinones, sterols, steroids, tannins, triterpenes (amyrins), and terpenoids [33,87]. *Jasminum brevilobum*’s leaves contain sesquiterpenoids (jatamansone) [35]. Leaves and flowers of *Jasminum fluminense* contain alkaloids, flavonoids, glycosides, phenols, saponins, triterpenes (squalene), and tannins [88]. *Jasminum grandiflorum* contains flavonoids (rutoside), monoterpenoids (geraniol, iridoids, secoiridoids), phenols (cresol), phenylpropanoid (eugenol), sesquiterpene alcohol (farnesol), phenolic acids, tannins, and terpenes [19,38,89]. *Jasminum nervosum* leaves contain alkaloids and flavonoids [89].

*Jasminum officinale* contains secoiridoid glycosides (aucubin, jasgranoside B, ligstroside, loganin, oleoside, oleuropein, and 8-dehydroxy shanzhiside), and flavonoid glycosides (sulfurein) [90,91,92], with leaves of *Jasminum officinale* containing phenols, phenylethanoids, flavonoids, and polyphenols [93,94]. Further, its stem contains sesquiterpenoids [95]. *Jasminum polyanthum’s* leaves and flowers contain alkaloids, phenols, quinines, saponins, and terpenoids [20]. Finally, leaves of *Jasminum syringifolium* contain alkaloids, flavonoids, triterpenoids, steroids, and tannins [43].

## 6. Antioxidant Potential of *Jasminum* spp.

In Table 2, the antioxidant potential of *Jasminum* plants is shown. The ethanol extract from the leaves of *J. abyssinicum* possessed strong antioxidant activity with IC_50_ 26.3 μg/mL, which was higher than the standard Trolox (IC_50_ 5.8 μg/mL) as per DPPH assay, whereas it showed an ORAC value of 1023.7 μg TE/mg extract. Moreover, a moderate amount of total phenolic content (401.3 μg GAE/mg) was also observed in the *J. abyssinicum* leaves extract by using the total phenolic content assay [96]. The study conducted by Moe et al. [30] demonstrated the antioxidant potential of ethanolic extract from *J. sessiliflorum* leaves and stems (0.5 mg/mL) by using DPPH, NO, and superoxide radical-scavenging assays as well as by measuring total phenolic content (TPC). In this study, ascorbic acid was used as a standard for DPPH (84.78%) and NO (78.96%) assays, and Gallic acid was used against superoxide (83.24%) radicals. The study revealed that the extract from leaves showed 11.12%, 51.49%, and 51.29% inhibition of DPPH, NO, and superoxide radical-scavenging activity, respectively, while the extract from stems showed only superoxide radical-scavenging activity with a 53.93% inhibitory rate. Also, the total phenolic content observed by leaves and stem extract was 2.09 and 23.23 mg GAE/g, respectively. Dose-dependent (25–400 µg/mL) antioxidant activity of ethanol, chloroform, and petroleum ether leaves extract of *J. arborescens* was observed by Bhagath et al. [97] with DPPH inhibition ranging from 40–90% and reducing power activity ranging from 0.2–0.45 absorbance at 700 nm. The maximum effect was found in ethanol extract, preceded by chloroform, and petroleum ether extract.

In a subsequent study, the ethanol extract of *J. auriculatum* leaves showed DPPH scavenging activity with an IC_50_ value of 33.39 μg/mL and total phenolic content of 8.47 mg GAE/g, whereas the standard, ascorbic acid, showed an IC_50_ value of 35.41 μg/mL in DPPH scavenging assay [32]. Boiling water (BWE), and hydromethanolic (HME) extracts of *J. grandiflorum* flower buds revealed an antioxidant effect, as evaluated using DPPH, superoxide, nitric oxide, and hydroxyl peroxide scavenging activity. The standard ascorbic acid exhibited IC_50_ > 6.93, 372.85, 248.25, and > 24.78 μg/mL, respectively. It was found that BWE displayed IC_50_ values of 327.89 and 38.27 μg/mL in superoxide and nitric oxide assays, respectively, whereas HME showed IC_50_ values of 1354.30 and 225.51 μg/mL. Similarly, both extracts, viz. HME (IC_50_189.93 μg/mL) and BWE (IC_50_ > 150.57 μg/mL) in DPPH assay were found to be less active than ascorbic (IC_50_ 6.93 μg/mL). However, BME showed an IC_50_ value of 397.09 μg/mL in hydroxyl peroxide radical scavenging activity which was almost similar to HME (IC_50_ 403.31 μg/mL [98]). Also, the ethanolic extract (JGLE) from leaves of *J. grandiflorum* displayed potent DPPH scavenging ability (IC_50_ 15 μg/mL) which was equivalent to ascorbic acid (IC_50_ 12 μg/mL). Moreover, JGLE also showed nitric oxide radical scavenging ability with IC_50_ 98 μg/mL compared to standard, curcumin (IC_50_ 92 μg/mL). Furthermore, JGLE increased reducing power with IC_50_ 19.5 μg/mL, where the IC_50_ value for standard quercetin was 15.5 μg/mL. In the superoxide anion assay, reduction of nitro blue tetrazolium (NBT) was found to rise in a dose-dependent pattern [99].

Likewise, Chaturvedi and Tripathi [100] inferred that the methanolic leaves extract of *J. grandiflorum* have strong antioxidant potential as evaluated by using iron-induced lipid peroxidation, reducing power, and trapped ABTS•+, superoxide, and superoxide radicals scavenging assays. The results showed that the extract showed ABTS•+ and superoxide scavenging activity with EC_50_ 222.50 and 207 μg/mL, respectively, where vitamin C (EC_50_ 36.72 μg/mL) was used as a standard for ABTS•+ assay. Moreover, the extract exhibited lower reducing capabilities at a concentration of 71.42 μg/mL compared to standard BHT (63.29 μg/mL) at 700 nm absorbance (Optical density 0.1). Concurrently, the extract inhibited iron-induced lipid peroxidation with EC_50_ 667.53 μg/mL, whereas the standards, BHT & quercetin, showed lipid peroxidation inhibition with EC_50_ 0.75 and 0.21 μg/mL, respectively. In the hydroxyl scavenging assay, the extract in the presence of EDTA scavenged hydroxyl radicals (non-site-specific reaction) with EC_50_ 288.19 μg/mL, while in the absence of EDTA (site-specific reaction) it showed EC_50_ 102.16 μg/mL. Along with this, the standard drug, BHT, showed EC_50_ 0.22 μg/mL for site-specific reaction and at 0.58 μg/mL for a non-site-specific reaction. Aqueous extract (500, 1000, 1500, and 2000 μg/mL) of *J. malabaricum* leaves, roots, and bark showed 7%, 22.2%, 44.4%, and 66.6% hydrogen peroxide scavenging activity, respectively, when compared with standard ascorbic acid (86%) [101].

The 90% methanolic and aqueous extracts of *J. mesnyi* leaves showed DPPH scavenging ability with IC_50_ 25.27 and 71.84 μg/mL, respectively, whereas the standard ascorbic acid and rutoside showed IC_50_ 8.84 and 3.78 μg/mL, respectively. Moreover, a concentration-dependent increase in reducing power was observed with both extracts (methanolic and aqueous) in the FRAP method. In addition, methanol extract, aqueous extract, and BHT (standard) displayed lipid peroxidation inhibitory activity with IC_50_ 84.69, 145.62, and 48.89 μg/mL, respectively [102].

The ethyl acetate and n-butanol fractions of methanolic leaves extract from *J. mesnyi* showed anti-oxidant potential using DPPH, nitric oxide (NO), and reducing power assay where standards, rutoside and ascorbic acid, were used. The n-butanol fraction showed high DPPH radical scavenging ability with IC_50_ 6.22 µg/mL compared to ascorbic acid (IC_50_ 6.54 µg/mL) and rutoside (IC_50_ 5.44 µg/mL), while the ethyl acetate fraction revealed an IC_50_ value of 153.45 µg/mL. Moreover, n-butanol and ethyl acetate fractions inhibited NO scavenging with IC_50_ 35.12 and 141.54 µg/mL, respectively, as compared to rutoside (IC_50_ 29.93 µg/mL) and ascorbic acid (IC_50_ 21.06 µg/mL). Furthermore, n-butanol at a concentration range of 25–400 µg/mL showed reductive capability with an absorbance range of 0.07–2.76 compared to ethyl acetate at 25–400 µg/mL (0.05–1.11) when compared to rutoside (0.06–2.76) and ascorbic acid with absorbance ranges between 0.07–2.82 [103].

Subsequently, a recent study of the 80% methanolic leaves extract from *J. multiflorum, J. azoricum, J. humile, J. officinale,* and *J. sambac* from two different locations (Arabian nights and Grand Duke of Tuskany) possessed DPPH radical scavenging activity with IC_50_ 34.8, 199.2, 94.6, 76.6, 130.7, and 155.5 μg/mL, respectively, while showing total phenolic amounts of 167.3, 56.9, 88.0, 133.4, 47.3, and 50.2 μg GAE/mg, respectively. Moreover, extract from *J. azoricum, J. officinale, J. multiflorum, J. humile,* and *J. sambac* (Arabian nights and Grand Duke of Tuskany) had total flavonoid contents of 46.3, 34.7, 44.4, 38.3, 39.2, and 40.5 μg QE/mg, respectively [94]. Guo et al. [104] showed that the isolated compounds Jasnervosides A-H isolated from stems of *J. nervosum* showed DPPH radical scavenging activity with inhibitory percentage ranges of 18.44 to 82.6%. Among the tested compounds, Jasnervosides A, B, D, and G exerted strong antioxidant activity with IC_50_ 0.22, 0.09, 0.19, and 1.21 μg/mL, respectively, whereas ascorbic acid showed IC_50_ 0.88 μg/mL.

*Jasminum nudiflorum* water-soluble and fat-soluble flower fractions showed ferric-reducing antioxidant power (FRAP) activity of 11.05 and 3.71 μmol Fe(II)/g, respectively, with total phenolic content of 2.42 and 0.66 mg GAE/g, respectively. Moreover, water soluble and fat-soluble flower fraction revealed trolox equivalent antioxidant capacity of 3.85 and 0.79 μmol trolox/g, respectively [105]. In another study, aqueous extract of *J. officinale* leaves displayed antioxidant potential with IC_50_ 41.16, 30.29, 20.19, and 29.48 µg/mL by using DPPH, nitric oxide, superoxide, and ABTS•+ radical scavenging assays, respectively, with ascorbic acid as a standard (IC_50_ 42.79, 36.74, 38.22, and 45.57 µg/mL, respectively). Moreover, the aqueous extract and ascorbic acid both showed a concentration-dependent reducing power (200–1000 µg/mL) as the absorbance increased with an increase in concentration by using reducing power assay [93]. Also, the 80% methanolic extract of *J. officinale* leaves displayed DPPH radical scavenging activity with IC_50_ value of 76.6 µg/mL [94], whereas *J. multiflorum* flower methanolic extract showed DPPH radical scavenging activity with IC_50_ value of 81 µg/mL [106]. Interestingly, the extract of *J. grandiflorum* dried flower buds proved to be a beneficial neuroprotective agent by acting on monoamine oxidase A (MAO-A), which catalyzes the reaction of monoamine deamination. Compared to the reference standard, clorgyline, *J. grandiflorum* extracts showed a higher MAO-A inhibiting activity, thereby supporting its antioxidant potential to alleviate symptoms of depression and lower cell oxidative injury [98].

Antioxidant activity is challenging to distinguish on basis of a single test model. Several in vitro methods that are used to assess the antioxidant effect of the desired samples such as DPPH radical scavenging assay, Hydroxyl scavenging assay, ABTS scavenging assay, Oxygen radical absorbance capacity (ORAC) LPO inhibition capacity (LPIC) assay, β-carotene–linoleic acid (linoleate) assay, and so forth. These test techniques differ from one another based on cost, accessibility, etc.

**Figure 4:**
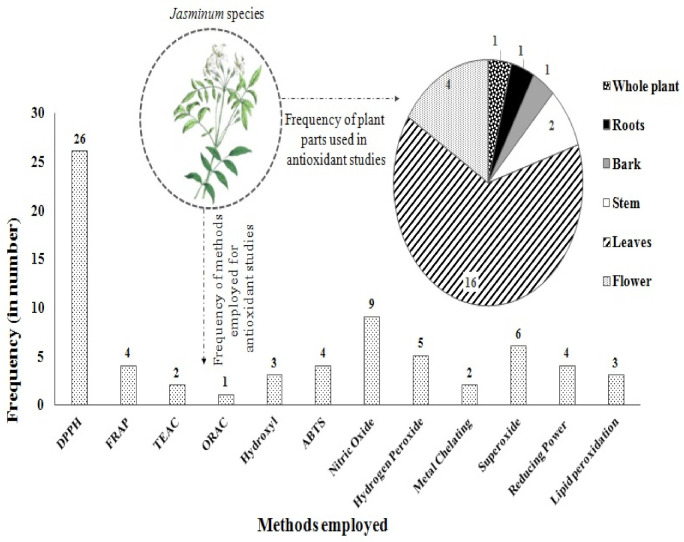
Frequency of method employed and plant parts used in antioxidant studies.

It is evident from Figure 4 that in in vitro study, four methods that are most frequently used are DPPH > Nitric oxide > superoxide radical > hydrogen-peroxide radical scavenging assay. On the basis of the most used method, phenylpropanoid glycoside (Jasnervoside B), isolated from the stems of *J. nervosum*, exhibited strong antioxidant potential with IC_50_ 0.09 µg/mL. Considerably, DPPH is considered the quickest, simplest, and rational approach out of all the in vitro methods, and thus it is used mainly for a sample’s antioxidant activity assessment. Further, Figure 4 shows that *Jasminum* spp. leaves have the highest frequency of plant parts used, followed by flower, stems, whole plant, and LRB (leaves, roots, and bark).

## 7. Oxidative Stress Related Diseases

Oxidative stress caused by ROS damages biomolecules (lipids, proteins, or DNA) thus contributing to cell survival regulation, inflammation, and stress responses [107,108,109]. Prolonged oxidative stress results in damage of body organs, which can potentially lead to the progression of chronic diseases like myocardial infarction, rheumatoid arthritis, diabetes, inflammatory diseases, cancer, vascular diseases, neurodegenerative diseases, and other metabolic diseases [110,111]. Enzymatic and non-enzymatic antioxidants provide a defense mechanism against free radicals by quenching or scavenging them from having harmful effects on the body. Catalase, thioredoxin, coenzyme Q, glutathione peroxidase, beta carotenoids, superoxide dismutase, polyphenols, glutathione, glutathione transferase, and glutathione reductase are widely evaluated antioxidants in the treatment of oxidative damage related diseases [112,113,114,115].

## 8. Impact of *Jasminum* Plants against Oxidative Stress In Vivo

The role of *Jasminum* spp. in combating oxidative stress related disorders is highlighted in Table 3. The anti-lipid peroxidative potential and chemopreventive efficacy of ethanolic extract (JgEt) from flowers of *J. grandiflorum* was evaluated on 7,12- enz(a)anthracene (DMBA; 25 mg, s.c.)-induced Wistar albino rat mammary carcinogenesis. The extract (300 mg/kg p.o.) completely prevented the occurrence of tumours, while preneoplastic lesions that were mild to moderate (hyperplasia, dysplasia, and keratosis) were found in histopathological evaluation of extract-treated rats. Moreover, JgEt significantly (*p* < 0.05) downregulated the levels of TBARS and improved the antioxidant status when compared with the DMBA-treated group. In addition, the extract markedly incremented (*p* < 0.05) in vitamin C level (in plasma), vitamin E level (in plasma and erythrocytes), and reduced glutathione level (in plasma and erythrocytes) with respect to the DMBA group. Also, superoxide dismutase (SOD), glutathione peroxidase (GPx), and catalase (CAT) levels were increased in mammary plasma, erythrocytes, and tissues of DMBA-treated rats, however, levels of vitamin E, glutathione peroxidase, and reduced glutathione were lowered (*p* < 0.05) in mammary tissue of experimental animals as compared to DMBA-treated rats. It was concluded that the extract showed chemopreventive efficacy in experimental mammary carcinogenesis [116]. Also, the hydromethanolic (HME) and boiling water (BWE) extracts of *J. grandiflorum* flower buds (dried) were assessed for in vitro efficacy towards central nervous system (CNS) disorders by measuring acetylcholinesterase (AChE), monoamine oxidase A (MAO-A), and butyrylcholinesterase (BuChE) inhibitory activity. It was observed that, both the extracts displayed MAO-A inhibitory activity with IC_50_ values of 603.16 μg/mL (HME) and 699.74 μg/mL (BWE) whereas the reference compound (clorgyline) showed an IC_50_ value > 0.012 μg/mL. Moreover, BWE, HME, and galantamine (reference compound) exhibited AChE inhibition with IC_25_ 1731.08, 1913.06, and 0.79 μg/mL, respectively. In addition, BuChE was highly inhibited by HME (IC_50_ 2610.87 μg/mL) followed by BWE (IC_50_ 5175.75 μg/mL) but weaker than reference compound, galantamine (IC_50_ 4.71 μg/mL). It was concluded from the study that the dried flower buds from *J. grandiflorum* can be used in treating psychiatric disorders and this activity is associated with antioxidant protection [98].

Similarly, Chaturvedi and Tripathi [100] showed that the methanolic leaves (100–800 μg/mL) extract of *J. grandiflorum* significantly (*p* < 0.001) inhibited LPS (20 ng/mL)-induced NO production in peritoneum fluid isolated macrophages from normal healthy Charles Foster (CF) strain albino rats in a concentration-dependent manner (300–800 μg/mL) with an inhibitory range of 9.5 to 4.41 μM/1 × 10^5^ cells as compared with an experimental control value (14.15 μM/1 × 10^5^ cells). Additionally, the wound healing effect of ointment (2% and 4%, topically) prepared using *J. grandiflorum* leaves methanolic extract was evaluated on cutaneous wound healing in diabetic Charles Foster (CF) strain albino rats. The extract (2% and 4%) significantly contracted wounds by 76.35% (*p* < 0.05) and 96.12% (*p* < 0.01), respectively, on day 12 as compared to the diabetic control group (62.94%). Moreover, the levels of total hydroxyl proline, hexosamine, protein, and DNA were all substantially higher (*p* < 0.01) at 4% ointment treatment, and a little less significant (*p* < 0.05) at 2% ointment treatment when compared to the diabetic control group. Furthermore, 4% of ointment-treated wounds displayed highly significant (*p* < 0.01) tensile strength on day 10 in comparison to the untreated wounds. Histopathological analysis revealed that ointment in a concentration-dependent manner (2% and 4%) increased fibrous tissue, collagen, and blood vessels. Also, ointment at 4% significantly led to the rise of SOD (*p* < 0.05), catalase (*p* < 0.001), and GSH (*p* < 0.05) content, whereas it decreased lipid peroxidation level (*p* < 0.05) in wound tissue with respect to the diabetic control group. Additionally, this ointment at 2% significantly (*p* < 0.05) effected only catalase level. Thus, it was concluded that the wound healing effect of ointment from *J. grandiflorum* leaves was through antioxidants [117]. The anti-inflammatory potential of the ethanol root extract (EJS; 100, 200, and 400 mg/kg, p.o.) of *J. sambac* was investigated using acute (carrageenan-induced paw edema), and sub-chronic (cotton pellet-induced granuloma) inflammation model of Charles Foster albino rats using diclofenac (10 mg/kg p.o) as a reference standard. It was found that, EJS (400 mg/kg) and standard significantly (*p* < 0.001) inhibited rat paw edema after 3, 4, and 6 h of treatment as compared to the untreated control. Moreover, EJS inhibited granuloma formation by 3.7%, 5.93%, and 33.58% at 100, 200, and 400 mg/kg, while diclofenac showed 43.40% inhibition in granuloma formation. In addition, EJS extract decreased AST (*p* < 0.05 and *p* < 0.05), ALT (*p* < 0.05 and *p* < 0.05), and lipid peroxidation (*p* < 0.05 and *p* < 0.01) levels, whereas it increased SOD (*p* < 0.05 and *p* < 0.01) and catalase (*p* < 0.001) in rats edematous tissue after acute and sub-chronic inflammation exposure, respectively in comparison to respective model groups. Also, EJS extract decreased lipid peroxidation (*p* < 0.001) levels whereas enhanced SOD (*p* < 0.05 and *p* < 0.05) and catalase (*p* < 0.01) in the serum of acute and sub-chronic inflammation model, respectively, with respect to their model group. Likewise, the standard, diclofenac, markedly (*p* < 0.05) reversed the altered parameters in serum and edematous tissue by both the models [118].

In another study, the anti-aging effect of *J. sambac* flower extract fermented with *Lactobacillus rhamnosus* (F-FEJS, 0.1%, 0.25%, 0.5%, 1.0%, 2.5%) was assessed in UVB (40 mJ/cm^2^) or H_2_O_2_ (200 μM) -induced aging in HS68 dermal fibroblast cells. It was observed that the extract (2.5%) significantly (*p* < 0.001) reduced intracellular reactive oxygen species production (ROS) stimulated by UVB or H_2_O_2_ as revealed by fluorescence microscopy and flow cytometry analysis. Moreover, the extract (2.5%) markedly decreased p53, p21 and p16 levels in H_2_O_2_ (*p* < 0.001, *p* < 0.001 & *p* < 0.001, respectively) and UVB treated HS68 cells when compared to H_2_O_2_ and UVB treated cells. In addition, the extract led to MMP-1 inhibition, SA-β-Gal positive cells, p-JNK, p-P38, p-ERK, and p-c-jun protein levels whereas efficiently upregulated collagen synthesis-related pathway components (p-smad2/3, TGF-β, COL3A1, and COL1A1), p-Nrf2 and antioxidant gene expression (HO-1) levels with respect to H_2_O_2_/UVB treated cells. Likewise, the extract enhanced p-Nrf2 nuclear translocation while down-regulated p-c-jun in the nuclear fractions. Further, it was confirmed that *J. sambac* flower extract attenuated H_2_O_2_/UVB-induced aging, ROS production, and degradation of collagen in HS68 cells through smad2/3, Nrf2, and c-jun pathways [119].

With a detailed understanding of the role of diseases generated by oxidative stress, *Jasminum* provided a useful approach in relation to their respective criteria for possible interventions in diseases related to oxidative stress (Table 3). Sengar et al. [118] scientifically validated the anti-inflammatory effect of *J. sambac* ethanol root extract against acute and chronic inflammation models with respect to their reference standard, diclofenac. The results suggested that the diclofenac illustrated efficient restoration of altered biochemical parameters in both acute and sub-chronic models’ edematous and granulomatous tissues than the plant extract. This plant has been used since ancient times as an anti-inflammatory, anti-pyretic, and anti-nociceptive agent [120,121]. Also, *J. sambac* leaves have been studied for their anti-inflammatory [122] and analgesic properties [123]. Due to the obvious negative effects of non-steroidal anti-inflammatory medicines (NSAIDs) and opioids, there is a strong demand for new products with minimal or no side effects and medicinal plants such as *Jasminum* will play a crucial role in this context.

*Jasminum* plants demonstrated protective activity against oxidative stress (triggered by free radicals) and its associated disorders. These plants significantly raise the proportion of antioxidant enzymes such as SOD, Gpx, and CAT and their effect is related to a broad range of bioactive constituents (Table 3). In the role of plants in combating the resistance section, the bioactive constituents of some *Jasminum* spp. reviewed in this study have already been discussed. In addition, antioxidant activity has been shown by some other plants, and their bioactive components are listed below. Leaves, bark, and roots from *Jasminum malabaricum* contain alkaloids, phenolics, glycosides, flavonoids, steroids, saponins, tannins, and terpenoids [101]. *Jasminum mesnyi* contains caffeic glycoside esters, flavonoids, sterols, secoiridoid glucosides (jasmoside, jasminoside), and triterpenes (amyrin) [124,125]. Similarly, leaves and flowers from *Jasminum multiflorum* contain alkaloids, cardiac glycosides, flavonoids, phenols, sterols, terpenoids, and tannins [126]. *Jasminum nudiflorum’s* stems contain secoiridoid glucosides (jasnudiflosides and nudiflosides) [127], while its flowers contain phenols (epicatechin, gallic acid, and protocatechuic acid) [105]. Additionally, *Jasminum sambac* contain anthranils, sesquiterpenes (farnesol), alkaloids, flavonoids (hesperidin), terpenoids (oleanoic acid, geraniol), phenols, tannins, steroidal saponins (daucosterol) and sterols, monoterpenoids (iridoid glucosides (jasminin, sambacin, sambacoside A-G), geraniol), phenylpropanoid (eugenol), sesquiterpene alcohol (farnesol) [24,118,121,123,128,129].

## 9. Mechanistic Basis of ROS Neutralization

**Figure 5:**
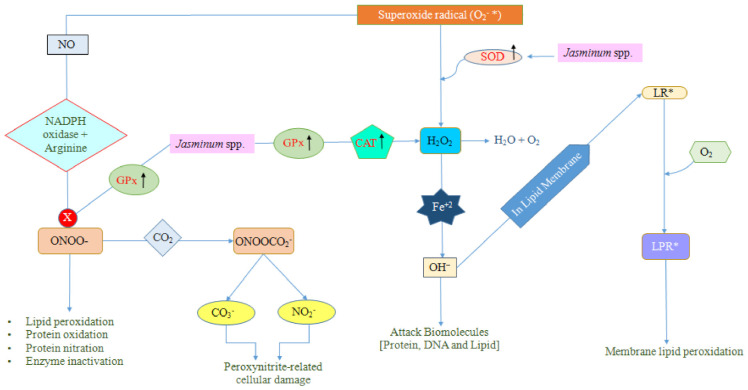
First line anti-oxidant defense mechanism mediated by *Jasminum* spp. to neutralize free radicals. Glutathione Peroxidase (GPx); catalase (CAT); Hydrogen peroxide (H_2_O_2_); Lipid Radical (LR*); Water (H_2_O); Oxygen (O_2_); Superoxide Dismutase (SOD); Lipid Peroxide radical (LPR*); Hydroxy radical (OH^−^); Fenton reaction (Fe^+2^); Nitric Oxide (NO); Carbon dioxide (CO_2_); Nicotinamide adenine dinucleotide phosphate oxidase (NADPH oxidase); Nitrosoperoxycarbonate (ONOOCO_2_^−^); Peroxynitrate (ONOO^−^); Carbonate (CO_3_^−^); Nitrogen dioxide radical (NO_2_^−^).

Adenosine triphosphates (ATPs), the energy currencies of the cell, are generated by mitochondria. Some low-energy electrons are released near the nucleus during the energy conversion, which are disposed of by the reduction of molecular oxygen to water, whilst a few of them escape and lead to the formation of superoxide radicals (O_2_^−^*) [130]. In a biological system, there are several different types of free radicals, but those generated from oxygen like superoxide anion (O_2_^−^*), singlet oxygen (O=O), are commonly referred to as reactive oxygen species (ROS). This superoxide anion can lead to the development of a variety of other reactive species like nitrosoperoxycarbonate, hydroxyl radical, peroxynitrite, and hydrogen peroxide through multiple chain reactions or pathways. However, certain key enzymes like glutathione peroxidase (GPx) and catalase (CAT), superoxide dismutase (SOD) assist to break down these free radicals into harmless and less active molecules (hydrogen peroxide/alcohol and O_2_) [130,131,132,133,134,135]. Among all, SOD is a key player for radical neutralization (Figure 5).

SOD is the first line defensive enzyme that assists in the dismutation of superoxide radicals into oxygen and hydrogen peroxide. In contrast, some of the hydrogen peroxide molecules in the presence of reduced iron (Fe^2+^), in what is called a Fenton reaction, are reduced into the deleterious (OH^−^*)* hydroxyl radical [135]. The hydroxyl radical is one of the highly reactive radicals which can result in cell toxicity [136,137]. CAT, GPx, and other enzymes prevent the formation of hydroxyl radical (OH^−^), by degrading the hydrogen peroxide (H_2_O_2_) into O_2_ and H_2_O. Sometimes, the hydroxyl radical facilitates the formation of lipid radicals (LR*) by acting on the lipid membrane, which further leads to the formation of lipid peroxy radical (LPR*) in combination with oxygen. This can result in attenuation of membrane-bound enzymes activity [138], dysfunction of membrane receptors [139], altered membrane permeability [140], as well as enhancing the rigidity of the membrane while lowering its fluidity [141]. On the other hand, multifaceted antioxidant enzyme, GPx is known to act in the removal of peroxynitrite anion, hydrogen-peroxides, and lipid-peroxides [142]. In the presence of NADPH-oxidase enzyme and arginine, superoxide anion and nitric oxide leads to the generation of peroxynitrite anion which is a powerful tissue-damaging oxidant. Furthermore, the peroxynitrite anion may often react with carbon dioxide, resulting in the formation of nitrosoperoxycarbonate, which gradually disintegrates to form nitrogen dioxide and carbonate radicals [143].

SOD, GPx, and CAT are imperative antioxidant enzymes that are crucial against the protection of the bio-system from free radicals. Hence, the proposed protective mechanisms of *Jasminum* plants explained in Figure 5 against free radicals might be that *Jasminum* plants would up-regulate the levels of the antioxidants enzymes which would further block the formation of the peroxynitrite anion or disintegrate hydrogen peroxide into water and oxygen.

## 10. Conclusions and Perspectives

In conclusion, most bio-activities were determined by the researchers, primarily for crude undefined extracts and the majority of the tests were conducted in vitro. In addition, for oxidative stress associated disorders, only 2 *Jasminum* species (*Jasminum grandiflorum* L. *Jasminum sambac* (L.) Aiton) have been studied (in vivo), yet they are highly successful in normalising various elevated parameters. The chemical profile of the *Jasminum* species revealed the presence of alkaloids, flavonoids, tannins, sterols, phenols, terpenoids, cardiac glycosides, terpenes, and secoiridoid glucosides. No studies have investigated the integrated role of *Jasminum* with standard drugs. It is expected that the data collected will serve as a useful protocol for researchers of herbal drug industry worldwide to explore various *Jasminum* species and their active components against human disorders. This review will provide more insight into the development of an effective drug candidate against diseases associated with oxidative stress and also against microbial diseases.

## Figures and Tables

**Table 1:** Antimicrobial potential of *Jasminum* spp.

Botanical Name	Extract/Solvent (Conc.)	Microbes	ZOI (mm)/MIC (μg/mL)	References
*Jasminum abyssinicum* Hochst. ex DC.	Aerial parts extract/Methanol (250–2000 μg/mL) Positive control	*Staphylococcus aureus* *Streptococcus pyogenes* *S. pneumonia* *Neisseria gonorrhoea* *Escherichia coli* *Bacillus cereus* *Shigella dysenteriae* *S. flexineri* *Salmonella typhi* *S. typhimuriumAspergillus flavus* *A. niger* *Candida albicans* *Trichophyton mentagrophytes* *T. violacum* *Cryptococcus neoformas* (Tetracycline, Co-trimoxazole, Gentamycin, Chloroamphenicol, Sulphadaizine, Cephalotin)	N.A. N.A. N.A. Active N.A. N.A. N.A. N.A. N.A. N.A. N.A. N.A. N.A. N.A. N.A. N.A. D.N.S.	[27]
	Leaves extract/Ethanol Positive control	*Bacillus cereus* *Clostridium perfringens*, *Listeria monocytogenes* *Staphylococcus epidermidis* *Enterococcus faecalis* *Staphylococcus aureus* *Streptococcus pyogenes* *Bacteroides fragilis* *Escherichia coli*, *Pseudomonas aeruguinosa* *Salmonella enteritidis* *Candida albicans* (Ciprofloxacin, Tioconazole, Penicillin)	**MIC** 512 N.A. 512 512 N.A. N.A. 256 N.A. N.A. N.A. N.A. N.A. 0.015–8	[28]
*Jasminum angustifolium* (L.) Willd.	Flower extract/ Methanol (500 ppm) Positive control	*Bacillus* sp. *Escherichia coli* *Staphylococcus* sp. *Klebsiella pneumoniae* *Lactobacillus* sp. *Yersinia* sp. *Enterococcus* sp. *Pseudomonas* sp.	**ZOI** N.A. N.A. N.A. N.A. 6 5.5 5 6 D.N.S.	[29]
*Jasminum angustifolium* var. *sessiliflorum* (Vahl) P.S.Green	Leaves and Stem extracts/Ethanol (25 mg/50 µL) Positive control	*Escherichia coli* *Pseudomonas aeruginosa* *Staphylococcus aureus* *Enterococcus faecalis* *Bacillus cereus* *Candida albicans* [Chloramphenicol (30 μg/well)]	**ZOI** S: N.A; L: N.A S: 22; L: 17 S: 12; L: N.A S: 14; L: 11 S: 12; L: 11 S: 13; L: 15 24–30 except *Pseudomonas aeruginosa*	[30]
*Jasminum arborescens* Roxb.	Leaves extract / Methanol (50 mg/mL) Positive control	*Escherichia coli* *Pseudomonas aeruginosa* *Staphylococcus aureus* *Bacillus subtilis* [Streptomycin (1mg/mL)]	**ZOI** 2.8 3.1 3.7 3.6 2.8–3.6	[31]
*Jasminum auriculatum* Vahl	Leaves extract /Ethanol Positive control	*Bacillus subtilis*, *Staphyloccocus aureus* *Pseudomonas aeruginosa* *Micrococcus luteus* *Escherichia coli* *Aspergillius niger* *Candida albicans* (Ciprofloxacin against bacterial strains Fluconazole against fungi)	**MIC** 1560 6250 780 3125 12500 N.A. N.A. 1.25–2.5 2.5	[32]
*Jasminum azoricum* L.	Leaves extract/ Acetone (30 mg/mL) Positive control	*Staphylococcus aureus* *Bacillus cereus* *B. subtilis* *Escherichia col*i *Pseudomonas* sp.	**ZOI** 20 24 9 14 17 D.N.S.	[33]
	Flowers extract/Butanol (500 mg/mL) Positive control	*Salmonella typhi* *Staphylococcus aureus* *Pseudomonas* sp. *Vibrio cholerae* *Streptococcus* sp. *Corynebacterium* sp. *Enterobacter aerogenes* *Proteus vulgaris* *Escherichia coli* (Ampicillin)	22 15 20 18 17 14 N.A. 18 21 D.N.S	[34]
*Jasminum brevilobum* DC.	Leaves extract/Acetone, Water, Methanol, Petroleum ether, Jatamansone	*Staphylococcus aureus* *Bacillus subtilis* *Escherichia coli* *Klebsiella pneumoniae* *Proteus mirabilis* Positive control	**MIC** D: 0.44; E: 0.92; F: 1.17; G: 1.56; H: 0.05 D: 0.42; E: 0.62; F: 1.04; G: 1.36; H: 0.15 D: 0.89; E: 1.24; F: 1.09; G: 1.08; H: 0.07 D: 0.54; E: 0.66; F: 0.95; G: 1.00; H: 0.14 D: 0.49; E: 0.51; F: 0.60; G: 0.92; H: 0.09 D.N.S.	[35]
*Jasminum fluminense* Vell.	Root extracts/ Methanol Positive control	*Candida albicans* *Gardnerella vaginalis* *Neisseria gonorrhoeae* *Oligella ureolytica* (Ciprofloxacin)	**MIC** 3100 <12,500 6300 3100 10–< 10	[36]
*Jasminum grandiflorum* L.	Leaves extract/Aqueous and Ethanol (hot solvent) Positive control	*Streptococcus mutans* *Lactobacillus acidophilus* (Ciprofloxacin)	**MIC** J: 6.25 E: 50 J: 25 E: 50 10–< 10	[37]
	Plant extract/Ethanol (500 μg/μL) Positive control	*Enterococcus faecalis* *Hafnia alvei* *Pseudomonas aeruginosa* *Proteus vulgaris* *Plesiomonas shigelloides* *Staphylococcus epidermidis* *S. aureus,* *S. saprophyticus* *S. pyogenes* *Salmonella typhi* *Shigella flexneri* *S. sonnie* *S. boydii* *S. dysenteriae*	**ZOI** N.A. N.A. 7 N.A. N.A. 15 7 7 7 N.A. 10 7 7 6 D.N.S.	[38]
*Jasminum grandiflorum* subsp. *floribundum* (R.Br. ex Fresen.) P.S.Green	Plant extract/Methanol (10 mg/mL) Positive control	*Escherichia coli* *Proteus vulgaris* *Pseudomonas aeruginosa* *Staphylococcus aureus* *Sarcina lutea* *Bacillus subtilis* *Mycobacterium phlei* *Candida albicans* (Ofloxacin, Amphotericin B)	**ZOI** 14 12 22 20 20 15 N.A. 22 D.N.S.	[39]
*Jasminum nervosum* Lour. (Synonym *Jasminum subtriplinerve* Blume)	Stem and leaves extract/petroleum ether, ethyl acetate, ethanol, methanol and water Positive control	*Escherichia coli* *Pseudomonas aeruginosa* *Bacillus subtilis* *Staphylococcus aureus* *Aspergillus Niger* *Fusarium oxysporum* *Candida albicans* *Saccharomyces cerevisiae*	**MIC** F: 200 AE: N.A G: 100 I and J: 200 AE: N.A. AE: N.A. AE: N.A. AE: N.A. D.N.S.	[40]
	Leaves extract/ Methanol [80% methanol at a ratio of 1:5 (*v*/*v*, dry plant material/solvent)] Positive control	*Fusarium solani* *F. oxysporum* *Rhizoctonia solani*	N.A. N.A. Active D.N.S.	[41]
*Jasminum officinale* L.	Essential oil from flowers extract Positive control	*Trichosporon ovoides* [Imidazole (50 µg/disc) Nystatin B (100 µg/disc)]	**MIC** 3.1 12.5 6.2	[42]
*Jasminum polyanthum* Franch.	Flower and leaf extracts/ water extract (2 g flowers as well as leaves used for extract preparation) Positive control	*Escherichia coli* *Klebsiella pneumonia*e *Staphylococcus aureus* *Pseudomonas aeruginosa* *Aspergillus flavus* *A. niger* (Gentamicin for bacterial strains)	**ZOI** Fl: 8; L: 7 Fl: 9; L: 8 Fl: 13; L: 11 Fl: 13; L: 12 Fl: 8; L: 10 Fl: N.A; L: N.A 10	[20]
*Jasminum syringifolium* Wall. ex G.Don	Leaves extract/ Methanol (100 g leaves in 95% methanol) Positive control	*Escherichia coli* *Pseudomonas aeruginosa* *Staphylococcus aureus* *Bacillus cereus* *Staphylococcus epidermidis* *Vibrio cholerae* *Proteus mirabilis* *Shigella flexneri* *Salmonella enterica typhi* *Klebsiella pneumoniae* *Aspergillus niger* *Candida albicans* (Gentamycin for bacterial strains Nystatin for fungi)	**ZOI** 21.33 16.67 21.67 22.33 16.33 18.67 15.33 22.67 19.33 18.33 17.33 15.33 12.67–22.67 17.67–21.33	[43]

S: Stem; L: Leaves; Fl: Flower; D: Acetone; E: Water; F: Methanol; G: Petroleum ether; H: Jatamansone; I: Ethyl acetate; J: Ethanol; AE: All extracts (petroleum ether, ethyl acetate, ethanol, methanol and water extracts), N.A.: Not active; ZOI: Zone of inhibition; MIC: Minimum inhibitory concentration; D.N.S.: Data not shown.

**Table 2:** In vitro antioxidant activity of *Jasminum* spp.

Botanical Name	Part Used	Solvent/Compound/Conc.	Method Used and Major Findings (IC_50_ and EC_50_- μg/mL)	References
*Jasminum abyssinicum* Hochst. ex DC.	L	E	DPPH (IC_50_) = 26.3 ORAC = 1023.7 μg TE/mg	[96]
*Jasminum angustifolium* var. *sessiliflorum* (Vahl) P.S. Green (Synonym: *Jasminum sessiliflorum*)	L S	E (0.5 mg/mL) E (0.5 mg/mL)	DPPH = 11.12% NO = 51.49% O^−^_2_ = 51.29% O^−^_2_ =53.93%	[30]
*Jasminum arborescens* Roxb.	L	E, CH and PE (0.025–0.4 mg/mL)	DPPH= 40–90% Fe ^+3^ reducing power (absorbance at 700 nm) = 0.2 to 0.45	[97]
*Jasminum auriculatum* Vahl	L	E	DPPH (IC_50_) = 33.39	[32]
*Jasminum azoricum* L.	L	80% M	DPPH (IC_50_) = 199.2	[94]
*Jasminum grandiflorum* L.	F	BWE HME	DPPH (IC_50_) = 150.57 O^−^_2_ (IC_50_) = 327.89 NO (IC_50_) = 38.27 H_2_O_2_ (IC_50_) = 397.09 DPPH (IC_50_) = 189.93 O^−^_2_ (IC_50_) = 1354.30 NO (IC_50_) = 225.51 H_2_O_2_ (IC_50_) = 403.31	[98]
	L	E	DPPH (IC_50_) = 15 Reducing power (IC_50_) = 19.5 NO (IC_50_) = 98	[99]
	L	M	Iron-induced lipid peroxidation (EC_50_) = 667.53 ABTS•+ (EC_50_) = 222.50 O^−^_2_ (EC_50_) = 207 OH (EC_50_) = 288.19 (+EDTA) and 102.16 (−EDTA)	[100]
*Jasminum humile* L.	L	80% M	DPPH (IC_50_) = 94.6	[94]
*Jasminum malabaricum* Wight	L, R, B	Aq (500, 1000, 1500 and 2000 μg/mL)	H_2_O_2_ = 7, 22.2, 44.4, and 66.6%	[101]
*Jasminum mesnyi* Hance	L	EA (25–400 µg/mL) n-but (25–400 µg/mL)	DPPH (IC_50_) = 153.45 NO (IC_50_) = 141.54 FRAP= concentration-dependent Reducing power (absorbance range) = 0.05–1.11 DPPH (IC_50_) = 6.22 NO (IC_50_) = 35.12 FRAP= concentration-dependent Reducing power (absorbance range) = 0.07–2.76	[103]
	L	M Aq	DPPH (IC_50_) = 25.27 Lipid peroxidation assay (IC_50_) = 84.69 DPPH (IC_50_) = 71.84 Lipid peroxidation assay (IC_50_) = 145.62	[102]
*Jasminum multiflorum* (Burm.f.) Andrews	L	M	DPPH (IC_50_) = 34.8	[94]
	F	M	DPPH (IC_50_) = 81	[106]
*Jasminum nervosum* Lour.	S	Jasnervosides A * Jasnervoside B * Jasnervoside D * Jasnervoside G *	DPPH (IC_50_) = 0.22 DPPH (IC_50_) = 0.09 DPPH (IC_50_) = 0.19 DPPH (IC_50_) = 1.21	[104]
*Jasminum nudiflorum* Lindl.	F	Water-soluble (tetrahydrofuran) Fat soluble (methanol–acetic acid–water mixture; 0:3.7:46.3)	FRAP = 11.05 μmol Fe(II)/g TEAC = 3.85 μmol trolox/g FRAP = 3.71 μmol Fe(II)/g TEAC = 0.79 μmol trolox/g	[105]
*Jasminum officinale* L.	L	Aq	DPPH (IC_50_) = 41.16 NO (IC_50_) = 30.29 O^−^_2_ (IC_50_) = 20.19 ABTS•+ (IC_50_) = 29.48	[93]
	L	80% M	DPPH (IC_50_) =76.6	[94]
*Jasminum sambac* (L.) Aiton	F	M	DPPH (IC_50_) = 208	[106]
	L (Arabian nights) L (Grand Duke of Tuskany)	80% M 80% M	DPPH (IC_50_) = 130.7 DPPH (IC_50_) = 155.5	[94]

L: Leaves; R: Roots; B: Bark; S: Stem; F: Flower; WP: Whole plant; *: compound; E: Ethanol; Aq: Aqueous; M: Methanol; DPPH: DPPH radical scavenging activity; H_2_O_2_: Hydrogen peroxide radical scavenging activity; TEAC: Trolox equivalent antioxidant capacity; ABTS•+: ABTS radical scavenging activity; OH: Hydroxyl radical scavenging activity; NO: Nitric oxide radical scavenging activity; FRAP: Ferric reducing antioxidant power; O^−^_2_: Superoxide radical scavenging activity; ORAC: Oxygen radical absorbance capacity; CH: Chloroform; PE: Petroleum ether; BWE: Boiling water extract; EA: Ethyl acetate; n-but: n-butanol; HME: Hydromethanolic; +EDTA: In presence of Ethylenediaminetetraacetic acid (EDTA); - EDTA: In absence of EDTA, IC_50_: Half maximal inhibitory concentration; %: Percent scavenging.

**Table 3:** Effect of *Jasminum* spp. against oxidative stress-related diseases.

Botanical Name	Plant Part	Solvent/Dose	Activity	Model	Biomarkers Affected	References
*Jasminum grandiflorum* L.	F	E (300 mg/kg p.o.)	Chemo preventive	7,12-dimethylbenz(a)anthracene (DMBA)-induced rat mammary carcinogenesis	↑ vitamin E (plasma and erythrocytes) ↑ vitamin C (plasma) ↑reduced glutathione (plasma and erythrocytes) ↑ SOD, CAT (plasma, erythrocytes and mammary tissues) ↑ glutathione peroxidase (plasma, erythrocytes) ↓TBARS ↓ reduced glutathione (tissue) ↓glutathione peroxidase (tissue)	[116]
	L	M (100–800 μg/mL)	Anti-inflammatory	LPS (20 ng/mL)-induced nitric oxide in rat peritoneal macrophage	↓ NO production (13.26 μ M/1 x 10^5^ cells to 4.41 μM/1 x 10^5^ cells)	[100]
	L	O	Wound healing	Cutaneous wound healing in diabetic rats	↑ wound contraction ↑total hydroxyl proline, ↑ hexosamine ↑ protein ↑ DNA content ↑ Tensile strength ↑collagen & fibrous tissue ↑ number of blood vessels ↑SOD, CAT and GSH content ↓ lipid peroxidation	[117]
*Jasminum sambac* (L.) Aiton	R	E	Anti-inflammatory	Carrageenan-induced rat paw edema model and cotton pellet-induced granuloma in rats	↓ paw edema ↓ granuloma formation ↓ AST, ALT, LPO, ↑ SOD and CAT	[118]
	F	LFE	Anti-aging	UVB (40 mJ/cm^2^ ) or H_2_O_2_ (200 μM) -induced HS68 dermal fibroblast cell	↓ ROS production ↓ aging markers, such as p16, p21, and p53, ↓ MMP-1 ↓ SA-β-Gal -positive cells ↓ p-ERK, p-JNK, p-P38, and p-c-jun protein levels ↑ p-smad2/3 in the nuclear fraction ↑ TGFβ, p-smad2/3, COL1A1, and COL3A1 protein levels ↑ phoshpho-Nuclear respiratory factor 2 and antioxidant gene expression (HO-1)	[119]

F: Flower, R: Roots, L: Leaves, E: Ethanol, M: Methanol, BWE: a boiling water extract, HME: Hydromethanolic extract, LFE: *Lactobacillus rhamnosus* fermented extract, O: ointment, LPS: Lipopolysaccharide, UVB: Ultraviolet B, H_2_O_2_: Hydrogen peroxide, SOD: Superoxide Dismutase, CAT: Catalase, TBARS: Thiobarbituric acid reactive substances (lipid peroxidation), NO: Nitric Oxide, GSH: Glutathione, AST: Aspartate transaminase, ALT: Alanine transaminase, LPO: lipid peroxidation, ROS: Reactive Oxygen Species, MMP-1: Matrix metalloproteinase-1, SA-β-Gal: Senescence-associated beta-galactosidase; COL1A1: Collagen Type I Alpha 1; COL3A1: Collagen Type III Alpha 1, and TGF-β: Transforming growth factor-beta.
